# Abnormal emotional learning in a rat model of autism exposed to valproic acid *in utero*

**DOI:** 10.3389/fnbeh.2014.00387

**Published:** 2014-11-12

**Authors:** Anwesha Banerjee, Crystal T. Engineer, Bethany L. Sauls, Anna A. Morales, Michael P. Kilgard, Jonathan E. Ploski

**Affiliations:** School of Behavioral and Brain Sciences, University of Texas at DallasRichardson, TX, USA

**Keywords:** autism, learning, memory, valproic acid, amygdala, emotion, Pavlovian fear conditioning

## Abstract

Autism Spectrum Disorders (ASD) are complex neurodevelopmental disorders characterized by repetitive behavior and impaired social communication and interactions. Apart from these core symptoms, a significant number of ASD individuals display higher levels of anxiety and some ASD individuals exhibit impaired emotional learning. We therefore sought to further examine anxiety and emotional learning in an environmentally induced animal model of ASD that utilizes the administration of the known teratogen, valproic acid (VPA) during gestation. Specifically we exposed dams to one of two different doses of VPA (500 and 600 mg/kg) or vehicle on day 12.5 of gestation and examined the resultant progeny. Our data indicate that animals exposed to VPA *in utero* exhibit enhanced anxiety in the open field test and normal object recognition memory compared to control animals. Animals exposed to 500 mg/kg of VPA displayed normal acquisition of auditory fear conditioning, and exhibited reduced extinction of fear memory and normal litter survival rates as compared to control animals. We observed that animals exposed to 600 mg/kg of VPA exhibited a significant reduction in the acquisition of fear conditioning, a significant reduction in social interaction and a significant reduction in litter survival rates as compared to control animals. VPA (600 mg/kg) exposed animals exhibited similar shock sensitivity and hearing as compared to control animals indicating the fear conditioning deficit observed in these animals was not likely due to sensory deficits, but rather due to deficits in learning or memory retrieval. In conclusion, considering that progeny from dams exposed to rather similar doses of VPA exhibit striking differences in emotional learning, the VPA model may serve as a useful tool to explore the molecular and cellular mechanisms that contribute to not only ASD, but also emotional learning.

## Introduction

Autism Spectrum Disorders (ASD) are complex neurodevelopmental disorders characterized by repetitive behavior and impaired communication and social interactions. Despite these common areas of impairment that define ASD as a syndrome, the clinical presentation and severity of these core symptoms vary widely across ASD individuals. In addition to these core symptoms, ASD individuals often exhibit varying levels of intellectual functioning, with some ASD individuals exhibiting high IQs, while others exhibit profound intellectual disability (Miller and Ozonoff, 2000; Munson et al., 2008; Charman et al., 2011; Green et al., 2013b). Furthermore, a significant number of ASD individuals display maladaptive emotional responses. Numerous studies indicate that a subset of ASD individuals display higher levels of anxiety (Evans et al., 2005; Weisbrot et al., 2005; Gaigg and Bowler, 2007; Wolff and Symons, 2013) and impaired emotional learning (Gaigg and Bowler, 2007; South et al., 2011, 2012). A number of theories attribute this altered emotionality to abnormalities in brain networks that mediate social-cognitive processes such as face processing and empathy (Schultz, 2005; Bachevalier and Loveland, 2006; Chevallier et al., 2012). However the underlying mechanism causing such altered emotions remains largely unknown.

ASD is a highly heritable disorder (Bailey et al., 1995; Amir et al., 1999; Marshall et al., 2008; Hallmayer et al., 2011), but for most cases of ASD, it remains unclear which genes are important for the development of ASD and how these genes interact with environmental factors to induce ASD. Most of the existing animal models of ASD were created by targeting a single gene for disruption (Zhao et al., 2005; Adachi et al., 2009; Mao et al., 2009; Umeda et al., 2010). However; the recent upsurge of clinical cases of idiopathic ASD suggests that environmental teratogens could be an important factor in the development of ASD (Christianson et al., 1994; Moore et al., 2000; Rasalam et al., 2005) and, therefore, environmental models of ASD are an important tool for uncovering the neurobiological basis of ASD. One such model is the valproic acid (VPA) animal model of ASD (Rodier et al., 1997; Arndt et al., 2005; Kim et al., 2011). This model is based on the discovery that when the anticonvulsant drug, VPA, is administered to women during their first trimester of pregnancy, they are much more likely to have children with ASD and intellectual disability (Vorhees, 1987; Christianson et al., 1994; Kemper and Bauman, 1998; Moore et al., 2000; Rasalam et al., 2005). In this animal model, pregnant dams are administered a single dose of VPA on or around embryonic day 12.5 of gestation, during the time of neural tube closure. The resultant progeny display anatomical and behavioral abnormalities similar to human ASD (Rodier et al., 1996; Ingram et al., 2000; Schneider and Przewlocki, 2005; Schneider et al., 2008).

The core symptoms of ASD have been extensively studied using the VPA ASD animal model. Most of these studies examined progeny from dams that were exposed to either one of two different doses of VPA (500 and 600 mg/kg) (Schneider and Przewlocki, 2005; Schneider et al., 2007, 2008; Edalatmanesh et al., 2013; Kataoka et al., 2013; Kerr et al., 2013). A number of studies have examined how *in utero* exposure to VPA influences emotional learning. Of these studies, the most consistent observation is that progeny from dams exposed to 500 mg/kg of VPA on embryonic day 12.5 exhibit a reduction in fear extinction compared to progeny from saline exposed dams (Markram et al., 2008; Lin et al., 2013; Wang et al., 2013). These three studies and an additional study that examined progeny from dams exposed to VPA 600 mg/kg (Sui and Chen, 2012) also indicate that fear learning may be enhanced due to *in utero* VPA exposure. However, variability in the exact nature of the findings these studies report indicates that the VPA induced fear learning phenomenon may be more inconsistent or a less robust phenomenon compared to the reduced fear extinction phenomenon.

Because the VPA ASD animal model provides a unique avenue to examine the molecular basis of emotional learning and autism, we began to examine emotional learning using this model. We found that progeny from dams exposed to VPA 600 mg/kg exhibited impaired fear learning. This was a replicable phenomenon that we reproduced across cohorts of animals. When we examined progeny from dams exposed to VPA 500 mg/kg, we observed that these animals exhibited reduced fear extinction, which is consistent with previous findings (Markram et al., 2008; Lin et al., 2013; Wang et al., 2013). These are intriguing results in part because the 500 and 600 mg/kg doses of VPA are very similar. It has been shown previously that there is a dose dependent difference in fetal reabsorption when dams are administered VPA at 500 and 600 mg/kg dose respectively, indicating that these doses differ substantively in how they influence development (Favre et al., 2013). Interestingly some studies have found that ASD individuals have a reduced ability to be fear conditioned (Gaigg and Bowler, 2007; South et al., 2011, 2012), therefore indicating that the VPA 600 mg/kg dose may arguably be a more relevant dose to use for examining the biological basis of autism. Here we describe our results and discuss our findings.

## Methods

### Subjects

Sprague Dawley rats (*Charles River Laboratories*) were maintained on a 12 h light/dark cycle. Food and water were provided *ad libitum* throughout the experiment. Animal use procedures were in accordance with the National Institutes of Health Guide for the Care and Use of Laboratory Animals and were approved by the University of Texas at Dallas Animal Care and Use Committee. To obtain progeny exposed to either VPA or saline *in utero*, rats were mated overnight and pregnancy was determined by the presence of a vaginal plug (E1). Female rats were used only once for breeding. Male rats were used twice for breeding, but each female the male was mated with, were placed in different experimental groups. VPA was dissolved in 0.9% saline at a concentration of 250 mg/ml and the dams were given a single intraperitoneal injection of either 600 mg/kg of VPA in 0.9% saline (VPA-Hi), 500 mg/kg of VPA in 0.9% saline (VPA-Lo), or 0.9% saline alone on day E12.5 of pregnancy as previously described (Schneider and Przewlocki, 2005; Markram et al., 2008). All dams were housed individually and left undisturbed until they gave birth. The offspring were weaned on postnatal day (PD) 21 and animals of either sex were group housed (3–4 animals). Male offspring from a total of 30 saline litters (138 male rats), 31 VPA-Hi litters (91 male rats), and 12 VPA-Lo litters (77 male rats) were used for behavior experiments (PD65-90). At PD60, the animals were individually housed and handled for 5 min per day for 4 days and on the 5th day behavioral experimentation began. Animals were housed individually for the duration of experimentation. The behavior experiments were performed on animals from multiple litters to limit the possibility that our results are due to natural behavioral variability that may occur between litters (Lazic and Essioux, 2013). Table S1 contains the litter and cohort data that were used for each experiment including the order of experiments performed on each cohort/litter. Experiments that involved subjecting rats to pain were performed after less stressful experiments (i.e., open field, object recognition). Litter survival rate was determined for cohorts 4, 5, 7, and 8 by determining the ratio of the total number of pups that reached the weaning age to total number of pups born per litter. Average litter size per cohort was calculated for cohorts 1–6, and was based on the final number of pups that were alive at weaning. No animals died prematurely following weaning and all animals post-weaning appeared healthy. Individual experiments were performed in a single session in a counterbalanced fashion with respect to control and treated animals.

### Auditory fear conditioning

A Coulbourn Instruments fear conditioning system with computer controlled shockers, USB cameras for video monitoring/video capture and FreezeFrame Software (Actimetrics) for unbiased behavioral analysis was used to auditory fear condition rats and to test for conditioned fear responses. *Training:* Rats were auditory fear conditioned with a single trial consisting of a 180 s acclimation period (pre-shock period) followed by the presentation of a 30 s, 5 kHz, 75 dB tone that co-terminated with a 1 s, 0.75 mA foot shock in a dimly lit training chamber. Animals remained in the training chamber for an additional minute following the delivery of the foot shock (post-shock period). During the last 50 s of this period, the freezing behavior of each animal was measured [*Post-shock freezing (PSF)*] and subsequently the animals were placed back into their home cages. *Short term memory (STM):* Animals were tested for retention of STM 3 h post fear conditioning in a novel context which had distinct tactile, olfactory and visual cues compared to the auditory fear conditioning training chamber. STM testing consisted of a 1 min acclimation period followed by the presentation of two (30 s, 5 kHz, 75 dB) tones with an inter-trial interval of 2 min. Following the second tone, animals remained in the box for an additional 2 min and were subsequently returned to their home cages. *Long term memory (LTM):* Animals were tested for long term memory (LTM) 24 h post auditory fear training in the same context where STM was tested. Testing consisted of a 2 min acclimation period followed by the presentation of 10 (20 s, 5 kHz, 75 dB) tones with an inter-trial interval of 2 min. After the presentation of the last tone, animals remained in the box for an additional 1 min and were subsequently returned to their home cages. *Auditory fear extinction:* Fear extinction was tested on days 4 and 5 following auditory fear conditioning, in the same chambers where STM and LTM were tested previously. Testing consisted of a 2 min acclimation period followed by the presentation of 10 (20 s, 5 kHz, 75 dB) tones with an inter trial interval of 2 min. After the presentation of the last tone, animals remained in the chamber for an additional 1 min and were subsequently returned to their home cages. All trials were recorded using Freeze frame software. All testing occurred in chambers that were not illuminated. The absence of any movement excluding respiration was recorded as a freezing response, which was calculated by the automated Freeze frame software.

### Foot shock sensitivity

To evaluate sensitivity to foot shock, animals were placed in a chamber with shock grids and exposed to different intensities of foot shock ranging from 0.1 to 1.0 mA. The lowest shock intensity at which the animals reacted by flinching or jumping was considered the threshold for shock sensitivity. Scoring of foot shock sensitivity was performed by two trained observers blind to the experiment.

### Hot plate pain sensitivity

The threshold for pain sensitivity was assessed using a hot-plate analgesia meter (ITC Life Sciences). The hot-plate was set to a temperature of 55°C and the latency to hind paw lick was measured by an observer blind to the experiment.

### Anxiety/locomotor behavior

General anxiety/innate fear and locomotor behavior were examined in an open field. The open field apparatus consisted of a wooden rectangular (1.2 × 1.2 m) box. The periphery of the box was designated as the outer 0.30 m region of the box and the center zone was designated as the 0.6 × 0.6 m square region at the center of the box. Each animal was placed in a corner of the box and allowed to freely explore the field for 8 min in low light (~100 Lux) while being video recorded using a USB camera. After the session, the animal was removed from the apparatus and returned to the home cage and the open field apparatus was cleaned with 10% ethanol and allowed to dry completely between trials. Total entries to the center of the open field, amount of time spent in the center, distance traveled in the center, and mean distance traveled for each animal were calculated by the automated behavioral tracking system, ANYmaze (Stoelting). The total distance traveled for each animal was used to compare overall locomotory behavior between the groups. Differences in open field center entries, center time and center distance traveled between the groups were used to determine if there were differences in anxiety/innate fear.

### Auditory tone threshold

Multi-unit responses to tones were collected from primary auditory cortex (A1) as in previous studies (Engineer et al., 2014a,b). Rats were anesthetized with 50 mg/kg pentobarbital and placed in a custom head holder that does not obstruct the ears in a double-walled sound chamber. Rats received supplemental doses of 8 mg/mL pentobarbital during the recordings in order to maintain a state of areflexia. EKG and pulse oximetry were used to monitor heart rate and oxygen saturation. Four parylene-coated tungsten microelectrodes (2 MΩ, FHC) were simultaneously lowered orthogonally into the cortex to record action potentials. In order to maximally activate contralateral (right) auditory cortex, the speaker (Tucker–Davis Technologies FF1 speaker) was positioned 90° left of the midline. Tucker–Davis Technologies neurophysiology hardware and software (RA16 and RX5; SigGen and Brainware) were used for stimulus presentation and data acquisition. Auditory cortex in the rat can be reliably located using the lateral suture and underlying blood vessels as landmarks. A1 is located ~1 mm dorsal to the horizontal portion of the suture and ~1.5 mm posterior to the vertical portion of the suture (Kilgard and Merzenich, 1999). As in our earlier studies, recordings were made in layers IV/V of primary auditory cortex, and the location of each site was chosen to avoid damaging blood vessels (Perez et al., 2013; Centanni et al., 2014). A1 sites were defined by the well-defined tonotopy and short latency responses (Polley et al., 2007). Tone frequency intensity tuning curves (1–32 kHz in 0.125 octave steps & 0–75 dB SPL in 5 dB steps) were recorded at each A1 site using an interstimulus interval of 0.5 s.

### Object recognition test

The object recognition task was conducted as described previously (Frick and Gresack, 2003; Fernandez et al., 2008). Briefly, this task consisted of a habituation phase, sample phase, and choice phase and each of these were conducted on separate days. During the habituation phase, animals were placed in an empty rectangular box (60 cm W × 60 cm L × 47 cm H) and allowed to freely explore for 5 min. Twenty-four hours later the sample phase of the experiment was conducted, where animals were placed in the same empty rectangular box and allowed to freely explore for 2 min, and then placed in a holding cage while two identical objects were placed in the left and right side (5 cm from the walls) of the box. Animals were then immediately returned to the box and allowed to freely investigate until they accumulated a total of 30 s exploring the objects. Contact with either object using front paws or nose was considered as exploration of the object. After 24 h, object recognition memory was examined during the choice phase of the experiment, using the same procedure as in sample phase except that a novel object was substituted for one of the familiar training objects. Novel object location was counterbalanced across animals. Time spent with each object was recorded using ANYmaze software (Stoelting). Animals inherently prefer to explore novel objects; thus, a preference for the novel object [more time than chance (15 s) with the novel object] indicates intact memory for the familiar object. The use of 30-s total exploration time rather than fixed trial duration minimizes confounding influences of group differences in activity.

### Social interaction

Social interaction between rats was measured using a modified version of protocol similar to one described previously (Golden et al., 2011; Green et al., 2013a). On Day 1, the subject rat, (VPA or saline exposed rat) was habituated to the empty social interaction apparatus (61 × 61 cm gray box) for 5 min. On day 2, a wired enclosure was placed in the corner of the social interaction box and a novel test rat was placed inside this enclosure. The subject rat was then introduced into the social interaction box and allowed to interact with the novel test rat for 10 min. This allowed the measurement of social behavior initiated by the subject rat only. The region surrounding the novel test rat chamber was marked as the social interaction zone. The amount of time spent in the social interaction zone sniffing and exploring the test rat, the latency to the first entry into the social interaction zone, and the longest visit to the social interaction zone were recorded by ANYmaze software and these were considered measures of social interaction behavior. Mean distance traveled in the apparatus measured the overall locomotory behavior of the animals.

### Statistical analysis

Results were expressed as mean ± standard error of the mean (SEM) in the text and figures. A One-Way ANOVA for repeated measurements were used to calculate the statistical significance for all fear conditioning experiments. A One-Way ANOVA was used to calculate the statistical significance for all litter survival rate differences among Saline, VPA-Lo and VPA-Hi groups. A student's *t*-test was used to compare means between groups. Differences with *p* < 0.05 were considered statistically significant.

## Results

### Progeny from VPA exposed dams exhibit a dose dependent effect on fear learning

**Figure 1:**
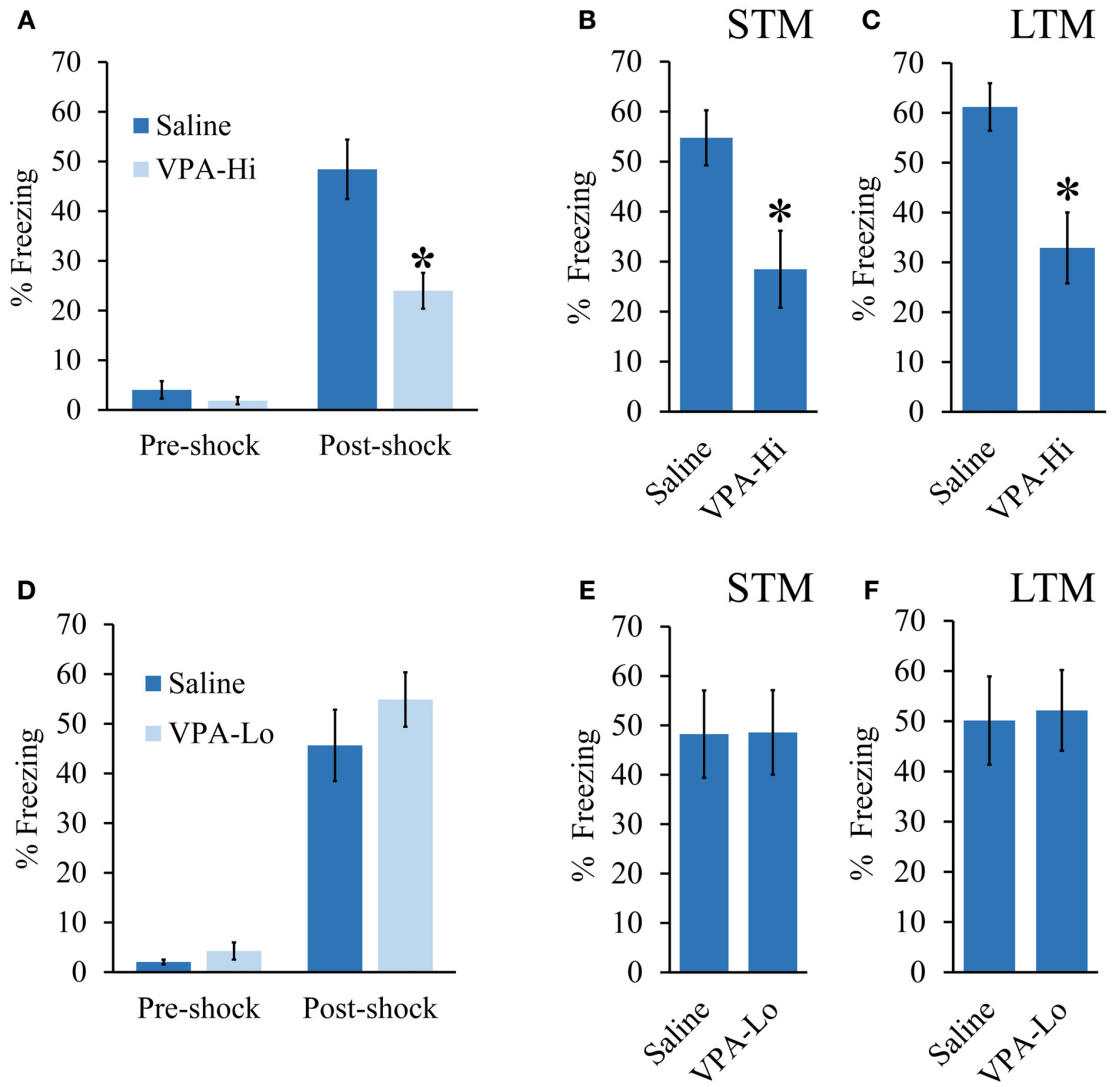
**Progeny from VPA exposed dams exhibit a dose dependent effect on fear learning: (A–C) Progeny from VPA-Hi and saline exposed dams were auditory fear conditioned (Saline = 16, VPA-Hi = 13)**. **(A)** Pre and post shock freezing was assessed immediately before and after exposure to tone-shock, respectively. **(B)** Auditory fear memory assessed 3 h post fear conditioning (i.e., STM) **(C)** Auditory fear memory assessed 24 h post fear conditioning (i.e., LTM). **(D–F)** Progeny from VPA-Lo and saline exposed dams were auditory fear conditioned (Saline = 12, VPA-Lo = 12). **(D)** Pre and post shock freezing was assessed immediately before and after exposure to tone-shock, respectively. **(E)** Auditory fear memory assessed 3 h post fear conditioning (i.e., STM) **(F)** Auditory fear memory assessed 24 h post fear conditioning (i.e., LTM). Bars represent the mean ± standard error of the mean (SEM) (^*^p < 0.05).

We investigated emotional learning using a Pavlovian auditory fear conditioning paradigm using progeny from dams treated with either 500 mg/kg (VPA-Lo) or 600 mg/kg (VPA-Hi) of VPA on embryonic day 12.5. In our first experiment, VPA-Hi and saline exposed animals were auditory fear conditioned to a 30 s, 5 kHz, 75 dB tone that co-terminated with a 0.75 mA foot shock. Pre-shock freezing levels between the VPA-Hi and saline exposed animals were not significantly different [*t*_(27)_ = 1.05, *p* = 0.30] (Figure 1A). The PSF levels were significantly higher in both groups compared to pre-shock freezing levels indicating both groups were conditioned [*t*_(24)_ = −6.00, *p* < 0.0001], however PSF levels were significantly lower in VPA-Hi exposed animals compared to saline exposed animals [*t*_(27)_ = 3.30, *p* = 0.002] indicating VPA-Hi exposed animals exhibited a reduced fear response either due to decreased acquisition of fear learning or inhibition of expression of fear (Figure 1A). Three hours following fear conditioning training, retention of STM was assessed by exposing the rats to two 5 kHz, 75 dB tones within a novel context and subsequently determining the freezing behavior of each rat, to each tone. The VPA-Hi animals exhibited significantly reduced freezing behavior as compared to the saline control group as revealed by a repeated measure ANOVA [*F*_(1, 27)_ = 8.129, *p* = 0.008] (sal = 16, VPA-Hi = 13) (Figure 1B). Twenty four hours following fear conditioning training, LTM was assessed in a similar manner as STM was, but freezing behavior was assessed during the presentation of ten, 5 kHz, 75 dB tones. The ANOVA indicated a significant effect for group [*F*_(1, 27)_ = 11.60, *p* = 0.002], where the VPA-Hi group exhibited significantly reduced freezing behavior compared to the saline control group (sal = 16, VPA-Hi = 13) (Figure 1C). These data collectively represent the combined data from two independent cohorts of animals that essentially produced the same results underscoring the reproducibility of these results. These data for each cohort are displayed separately in Image 1. Collectively VPA-Hi exposed animals exhibit impaired PSF, STM, and LTM relative to saline exposed animals.

Next, we examined VPA-Lo exposed animals for their ability to be auditory fear conditioned. VPA-Lo and saline exposed animals were fear conditioned and PSF, STM, and LTM were assessed in the same manner as mentioned above. VPA-Lo and saline exposed animals did not exhibit differences in pre-shock freezing levels [*t*_(22)_ = 0.84, *p* = 0.40] or PSF levels [*t*_(22)_ = 1.55, *p* = 0.13]. However both groups exhibited significantly higher PSF levels compared to pre-shock freezing levels [*t*_(22)_ = −9.79, *p* < 0.0001] (Figure 1D). These data indicate that acquisition of fear conditioning was normal in VPA-Lo exposed animals. Freezing levels during the STM test were measured and ANOVA revealed that the effect for group [*F*_(1, 22)_ = 0.001, *p* = 0.978], were not significant indicating that the freezing levels during the STM test were not different between the VPA-Lo and the saline control groups (sal = 12, VPA-Lo = 12) (Figure 1E). Twenty four hours after fear conditioning training, freezing levels during the LTM test were assessed. ANOVA indicated that freezing levels for the VPA-Lo and saline groups during the LTM test did not differ significantly as there was no effect for group [*F*_(1, 22)_ = 0.029, *p* = 0.86], (sal = 12, VPA-Lo = 12) (Figure 1F). Therefore in contrast to the VPA-Hi exposed animals that exhibited significantly impaired PSF, STM, and LTM relative to saline controls, VPA-Lo exposed animals did not differ in PSF, STM, and LTM relative to saline controls.

### Progeny from VPA-Lo exposed dams exhibit reduced fear extinction to a conditioned auditory cue

**Figure 2:**
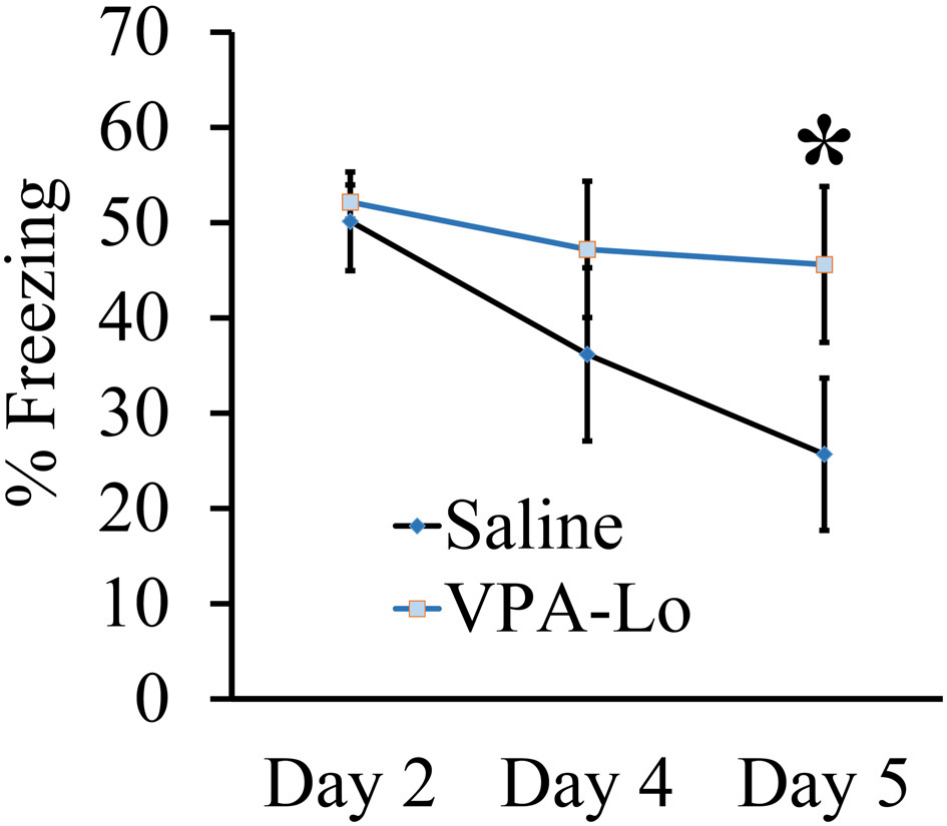
**Progeny from VPA-Lo exposed dams exhibit reduced fear extinction to a conditioned auditory cue**. Progeny from VPA-Lo and saline exposed dams were auditory fear conditioned and LTM/extinction learning was assessed on days 2, 4, and 5 post fear conditioning (Saline = 12, VPA-Lo = 12). Bars represent the mean ± s.e.m. (^*^p < 0.05).

Our previous experiment demonstrated that VPA-Lo exposed rats exhibit intact STM and LTM relative to saline controls, 3 and 24 h post fear conditioning respectively. However, a previous study indicated that rats exposed to 500 mg/kg (VPA-Lo) *in utero*, exhibit elevated conditioned fear memory, and reduced fear extinction (Markram et al., 2008; Lin et al., 2013; Wang et al., 2013). Therefore, in our next experiment, we wanted to determine if in our laboratory, VPA-Lo animals display reduced fear extinction. VPA-Hi exposed animals were excluded from this experiment since they failed to acquire normal levels of fear memory. To examine auditory fear extinction rates between VPA-Lo and saline exposed animals, we measured conditioned tone fear memory on day 4 and day 5 post fear conditioning by subjecting the rats to ten 20 s, 5 kHz, 75 dB tones in the same context used to examine LTM. A repeated measures ANOVA revealed no effect for group [*F*_(1, 22)_ = 1.12, *p* = 0.30], indicating that on day 4 there was not a statistically significant difference in extinction rates between the VPA-Lo and saline exposed animals. However on day 5 of extinction testing the difference in freezing between the VPA-Lo and saline groups became more striking, where a significant difference for increased freezing in VPA-Lo exposed animals relative to saline controls was found [*F*_(1, 22)_ = 7.26, *p* = 0.01], (sal = 12, VPA-Lo = 12) (Figure 2). These findings are consistent with the view that VPA-Lo exposed animals exhibit reduced fear extinction.

### The fear conditioning deficit observed in progeny from VPA-Hi exposed dams is likely not due to deficits in sensory modalities necessary for auditory fear conditioning

Progeny from VPA-Hi exposed dams exhibited a reduced ability to be auditory fear conditioned as indicated by reduced freezing relative to saline controls during PSF, STM, and LTM tests. However, these data could be a result of impairments in the neurobiological mechanisms that subserve learning or they could be due to sensory deficits in systems that are required for auditory fear conditioning, such as nociceptive or auditory modalities. Therefore we examined VPA-Hi and saline exposed animals for nociception and audition, using the hot plate test, shock sensitivity tests and by measuring neuronal spike firing within the auditory cortex in response to exposure to 5 kHz 75 dB tones, respectively.

**Figure 3:**
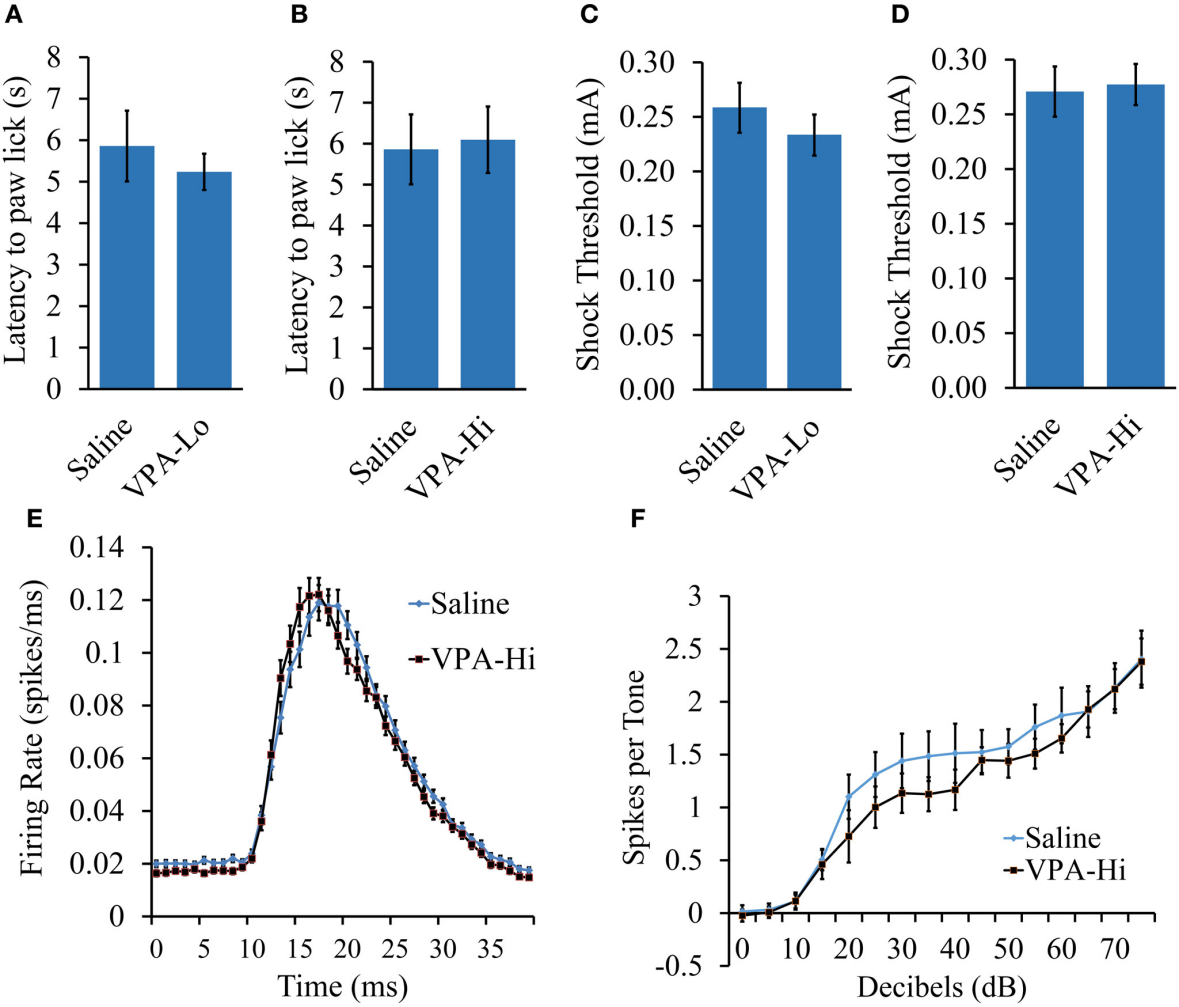
**The fear conditioning deficit observed in progeny from VPA-Hi exposed dams is likely not due to deficits in sensory modalities necessary for auditory fear conditioning**. **(A)** Thermal nociception as measured by the hotplate test did not differ between progeny from VPA-Lo and saline exposed dams (Saline = 12, VPA-Lo = 12) **(B)** or progeny from VPA-Hi and saline exposed dams (Saline = 12, VPA-Lo = 11) **(C)** Response to foot shock stimuli did not differ between progeny from VPA-Lo and saline exposed dams (Saline = 10, VPA-Lo = 10) **(D)** or progeny from VPA-Hi and saline exposed dams (Saline = 12, VPA-Lo = 11). **(E)** The primary auditory cortex response strength and response latency to a 5 kHz tone did not differ between progeny from VPA-Hi and saline exposed dams (Saline = 10, VPA-Hi = 10). **(F)** Hearing ability to a 5 kHz, 0–75dB tone did not differ between progeny from VPA-Hi and saline exposed dams (Saline = 10, VPA-Hi = 10) as measured by auditory cortex neuronal spike firing upon exposure to 5 kHz, 0–75 dB tones. Data presented as mean ± s.e.m.

VPA and saline exposed animals were examined for nociceptive ability using the hot plate test. Animals were placed on a 55°C hot plate and the latency for each animal to paw lick was measured. No significant difference in latency to paw lick was found in either VPA-Lo animals [*t*_(18)_ = 0.61, *p* = 0.54] (sal = 12, VPA-Lo = 12) or VPA-Hi animals [*t*_(18)_ = −0.19, *p* = 0.8] (sal = 12, VPA-Hi = 11), in comparison to saline control animals (Figures 3A,B). These data indicate that *in utero* exposure to VPA at both the high and low doses does not significantly impair nociception.

To measure the threshold at which progeny from VPA and saline exposed dams responded to foot shock stimuli, the animals were exposed to foot shocks ranging from 1.0 to 1.0 mA at an increment of 0.1 mA and the lowest shock intensity at which the animals reacted was considered the threshold for shock sensitivity. Sensitivity to foot shocks did not differ significantly in either the VPA-Lo group [*t*_(22)_ = 0.84, *p* = 0.40] (sal = 12, VPA-Lo = 11), or the VPA-Hi group [*t*_(21)_ = −0.12, *p* = 0.90] (sal = 10, VPA-Hi = 10), when compared to animals exposed to saline (Figures 3C,D). Overall, these data indicate that progeny from VPA-Hi, VPA-Lo and saline exposed dams exhibit similar levels of shock sensitivity and therefore the deficit in fear conditioning exhibited by the VPA-Hi group relative to saline controls is likely not due to an impaired ability to perceive the foot shock. Notably, the threshold for shock sensitivity for all groups was far below the shock intensity used for fear conditioning.

To determine whether VPA-Hi exposed animals possess impaired hearing to a 5 kHz, 75 dB tone, we compared the action potential spike firing of neurons within the primary auditory cortex (A1) from VPA-Hi and saline exposed animals. The average response amplitude evoked by tones within ¼ octave of 5 kHz was not significantly different in VPA-Hi rats compared to saline rats (1.26 ± 0.06 spikes/tone vs. 1.27 ± 0.07 spikes/tone, *p* = 0.94, Figure 3E). There was no significant difference in the peak firing latency to tones within ¼ octave of 5 kHz in VPA-Hi rats compared to saline rats (19.48 ± 0.3 ms vs. 20.02 ± 0.3 ms, p = 0.22, Figure 3E). The 5 kHz tone was presented at intensities ranging from 0 to 75 dB, and the average number of spikes per tone was plotted by tone intensity. The response strength to tones in A1 was unimpaired in VPA exposed rats compared to saline rats [*F*_(1, 14)_ = 0.64, *p* = 0.43] thereby supporting the notion that progeny from VPA-Hi exposed dams do not exhibit impaired hearing to a 5 kHz, 75 dB tone (Figure 3F).

### Progeny from VPA exposed dams exhibit increased anxiety and normal locomotor behavior

**Figure 4:**
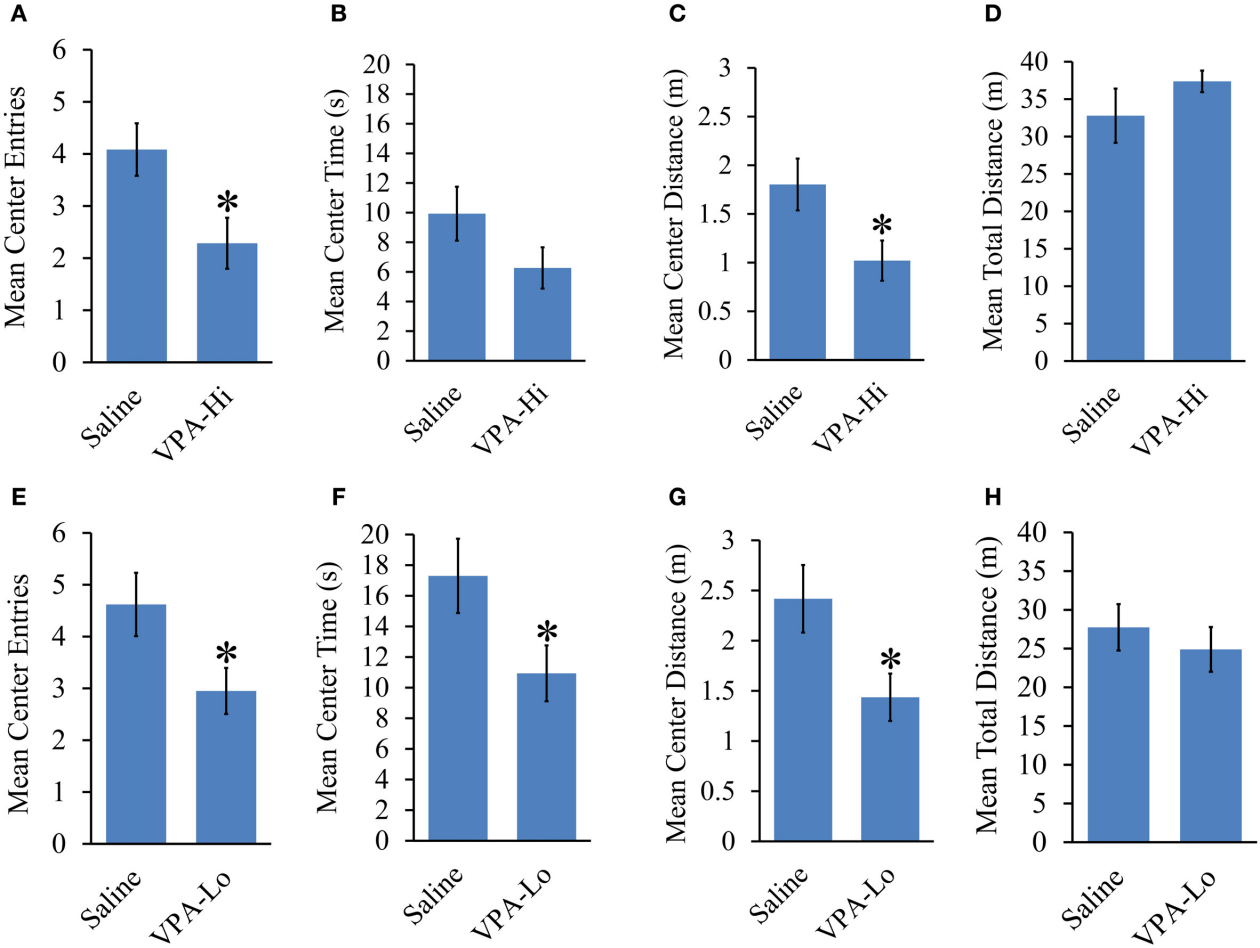
**Progeny from VPA exposed dams exhibit increased anxiety and normal locomotor behavior**. Progeny from VPA-Hi dams exhibit increased anxiety in an open field as indicated by, **(A)** a reduced number of center entries, **(B)** a trend of reduced center time, **(C)** a reduced center distance traveled, without affecting, **(D)** the mean total distance traveled as compared to saline exposed animals (Saline = 25, VPA-Hi = 21). Progeny from VPA-Lo exposed dams exhibit increased anxiety in an open field as indicated by, **(E)** a reduced number of center entries, **(F)** a reduced center time, **(G)** a reduced center distance traveled, without affecting, **(H)** the mean total distance traveled as compared to saline exposed animals (Saline = 21, VPA-Lo = 20). Data presented as mean ± s.e.m. (^*^p < 0.05).

The open field test was used to examine the relative level of anxiety in VPA and saline exposed animals. VPA-Hi exposed animals exhibited a reduced number of center entries [*t*_(43)_ = 2.42, *p* = 0.01], a trend of reduced center time [*t*_(43)_ = 1.54, *p* = 0.13] and reduced center distance traveled [*t*_(43)_ = 2.24, *p* = 0.03] when compared to saline animals during their 8 min exploration period in the open field, indicating an increased level of general anxiety (Figures 4A–C). However, the mean distance traveled [*t*_(43)_ = −1.09, *p* = 0.27], in the open field did not differ between VPA-Hi and saline exposed animals indicating that locomotor behavior was not significantly different between these groups (Figure 4D). VPA-Lo exposed animals also displayed a reduced number of center entries [*t*_(39)_ = 2.19, *p* = 0.03], a reduced center time [*t*_(39)_ = 2.08, *p* = 0.04] and reduced center distance traveled [*t*_(39)_ = 2.36, *p* = 0.02], compared to saline animals (Figures 4E–G). The mean distance traveled [*t*_(39)_ = 0.68, *p* = 0.49] in the field did not differ between VPA-Lo and saline exposed animals indicating that locomotor behavior was not different between these groups (Figure 4H). Collectively progeny from VPA exposed dams exhibit higher levels of anxiety as determined by open field behavior and these results are similar to what others have found, following both VPA-Lo (Markram et al., 2008; Lin et al., 2013) and VPA-Hi exposure (Schneider and Przewlocki, 2005; Schneider et al., 2007). For unequivocal demonstration that VPA exposure induces anxiety like behavior, these animals would also need to be assessed in the elevated plus maze task.

### Progeny from VPA exposed dams exhibit normal objection recognition memory

**Figure 5:**
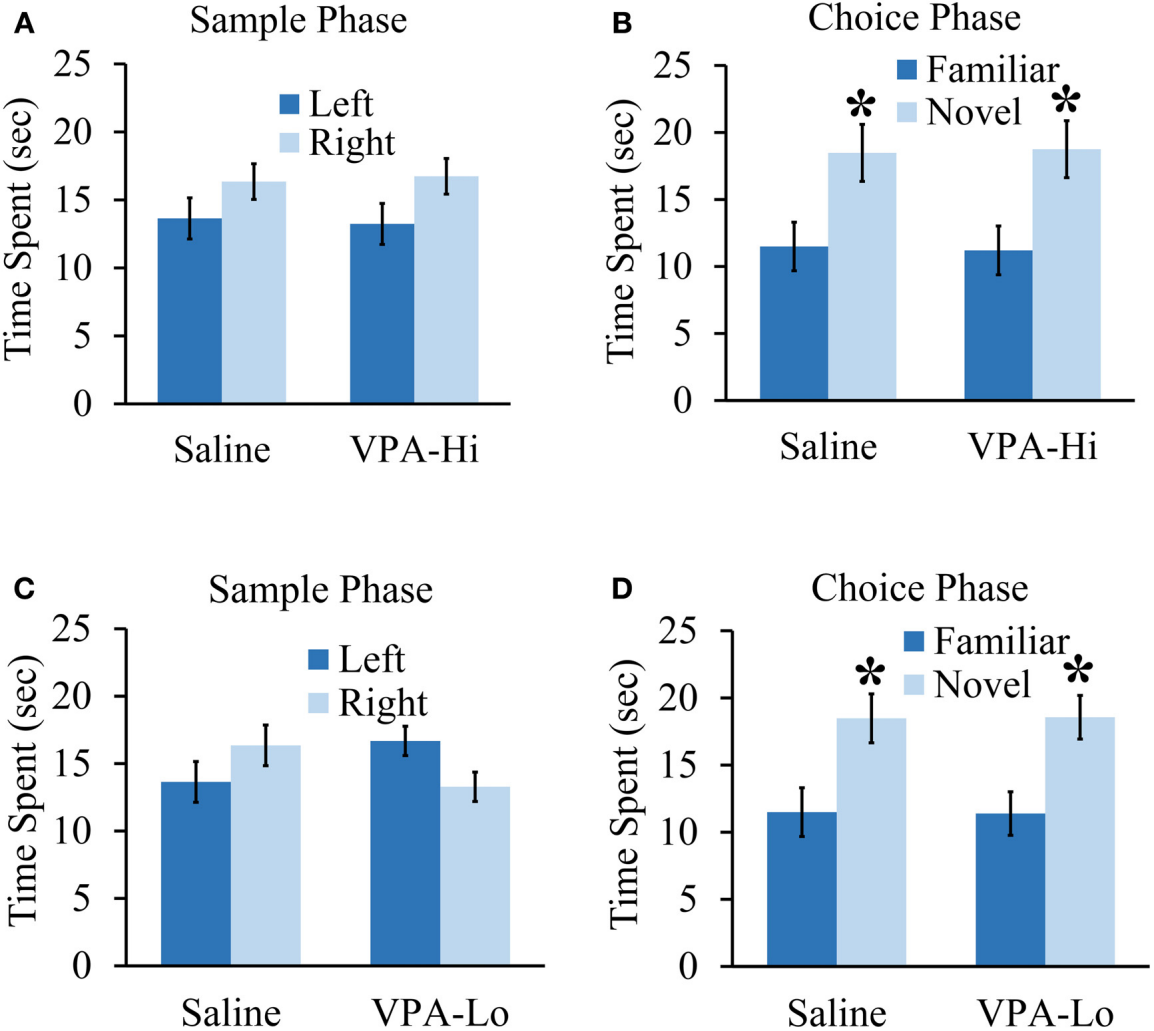
**Progeny from VPA exposed dams exhibit normal objection recognition memory**. **(A)** Progeny from VPA-Hi and saline exposed dams spent equal time with the familiar object during the sample phase of an object recognition test (saline = 10, VPA-Hi = 8). **(B)** VPA-Hi and saline animals spent more time than chance with a novel object during the choice phase of an object recognition test, indicating normal retention of object memory. **(C)** The VPA-Lo group had a slight preference for the object situated on left side compared to right side (saline = 10, VPA-Lo = 10) (saline = 10, VPA-Lo = 10). **(D)** VPA-Lo and saline exposed animals spent more time than considered chance with a novel object during the choice phase of an object recognition test, indicating normal retention of object memory. Data presented as mean ± s.e.m. time spent with each object (^*^p < 0.05).

In this experiment we wanted to determine if VPA exposed animals exhibit deficits in other forms of learning and memory in addition to deficits in associative fear learning. Specifically, we examined if VPA-Hi, VPA-Lo and saline exposed animals differ in their ability to form object recognition memory. During the sample phase, VPA-Hi and saline exposed animals were allowed to explore two identical objects situated on the left and right sides of the testing apparatus until they had accumulated 30 s of exploration time for these objects. Neither group exhibited within group differences for the amount of time the animals explored the left or right objects (VPA-Hi: *t*_(14)_ = −1.88, *p* = 0.08]; saline: [*t*_(14)_ = −1.26, *p* = 0.22]. Further, differences were not detected between VPA-Hi or saline exposed animals during the sample phase in the amount of time each group of animals explored the object located on the left side [*t*_(16)_ = 0.195, *p* = 0.84] and right side [*t*_(16)_ = −0.188, *p* = 0.85] (Figure 5A). Twenty-four hours later, object recognition memory was examined during the choice phase of the experiment by allowing the rats to explore an object identical to what was used during the sample phase (i.e., familiar object) and a novel object for 30 s. Both VPA-Hi [*t*_(14)_ = 2.51, *p* = 0.02] and saline [*t*_(18)_ = 2.718, *p* = 0.01] exposed animals spent significantly more time than chance exploring the novel object, indicating they both formed object recognition memory. Additionally, VPA-Hi and saline exposed animals did not spend significantly different amounts of time with the novel object [*t*_(16)_ = 0.09, *p* = 0.92] (Figure 5B).

Next, we examined VPA-Lo and saline exposed animals in their ability to form object memory. During the sample phase, VPA-Lo exposed animals exhibited a slight preference for the familiar object placed on the left side of the box compared to the right side [*t*_(18)_ = 2.21, *p* = 0.04]. However, differences were not detected between VPA-Lo or saline exposed animals during the sample phase in the amount of time each group explored the object located on the left side [*t*_(18)_ = −1.63, *p* = 0.11] and right side [*t*_(18)_ = −1.65, *p* = 0.11] (Figure 5C). Twenty-four hours later, object recognition memory was examined during the choice phase of the experiment by allowing the rats to explore an object identical to what was used during the sample phase (i.e., familiar object) and a novel object for 30 s. Both VPA-Lo [*t*_(18)_ = 3.12, *p* = 0.005] and saline [*t*_(18)_ = 2.71, *p* = 0.01] exposed animals spent significantly more time than chance exploring the novel object, indicating they both formed object recognition memory. Additionally, VPA-Lo and saline exposed animals did not spend significantly different amounts of time with the novel object [*t*_(18)_ = −0.03, *p* = 0.97] (Figure 5D). These data indicate that the VPA-Hi and VPA-Lo exposed animals exhibit normal object recognition memory relative to progeny from saline exposed dams. Collectively, our findings are consistent with other findings that progeny from VPA-Hi and VPA-Lo exposed dams do not exhibit deficits in object recognition memory (Schneider et al., 2007; Chomiak et al., 2014) or spatial memory (Markram et al., 2008; Bambini-Junior et al., 2011).

### Progeny from VPA-Hi exposed dams exhibit reduced social interaction

**Figure 6:**
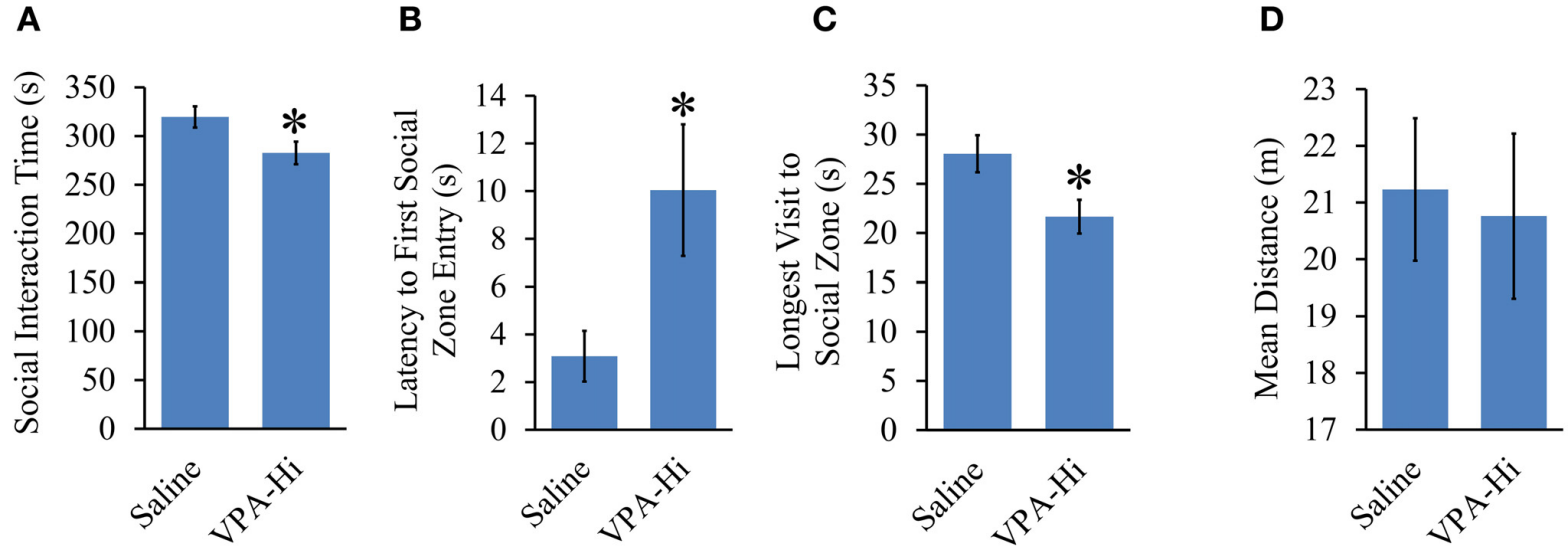
**Progeny from VPA-Hi exposed dams exhibit reduced social interaction**. **(A)** VPA-Hi animals spent less time in the social interaction zone compared to saline animals (saline = 15, VPA-Hi = 15). **(B)** Latency to first entry into the social interaction zone was higher in VPA-Hi animals compared to saline animals. **(C)** The longest visit to the social interaction zone was reduced in VPA-Hi animals. **(D)** Mean distance traveled in the social interaction apparatus did not differ significantly between the groups. Data presented as mean ± s.e.m. (^*^p < 0.05).

We found that VPA-Hi exposed animals exhibited reduced fear learning, and this was in sharp contrast to the results we obtained from VPA-Lo animals and one previous finding that reported that animals exposed to 600 mg/kg of VPA exhibited enhanced fear learning (Sui and Chen, 2012). Therefore to be sure something was not anomalous about our VPA-Hi animals, we examined if in our hands VPA-Hi animals also exhibit autistic-like behaviors similar to what others have reported previously (Schneider and Przewlocki, 2005; Lin et al., 2013). Specifically we examined the social interaction of VPA-Hi and saline control rats with a novel test rat. We observed that VPA-Hi animals spent less time in the social interaction zone [*t*_(28)_ = 3.47, *p* = 0.001], exhibited a higher latency of first entry into the social zone [*t*_(28)_ = −2.35, *p* = 0.02], and exhibited shorter visits to the social interaction zone [*t*_(28)_ = 2.51, *p* = 0.01] when compared to saline animals. The mean distance traveled in the social apparatus did not differ [*t*_(28)_ = 0.24, *p* = 0.80], between the groups (Figures 6A–D). These data indicate that VPA-Hi animals exhibit reduced social interaction as compared to saline animals.

### Litters from VPA-Hi exposed dams are smaller and VPA-Hi exposed progeny exhibit reduced survival

**Figure 7:**
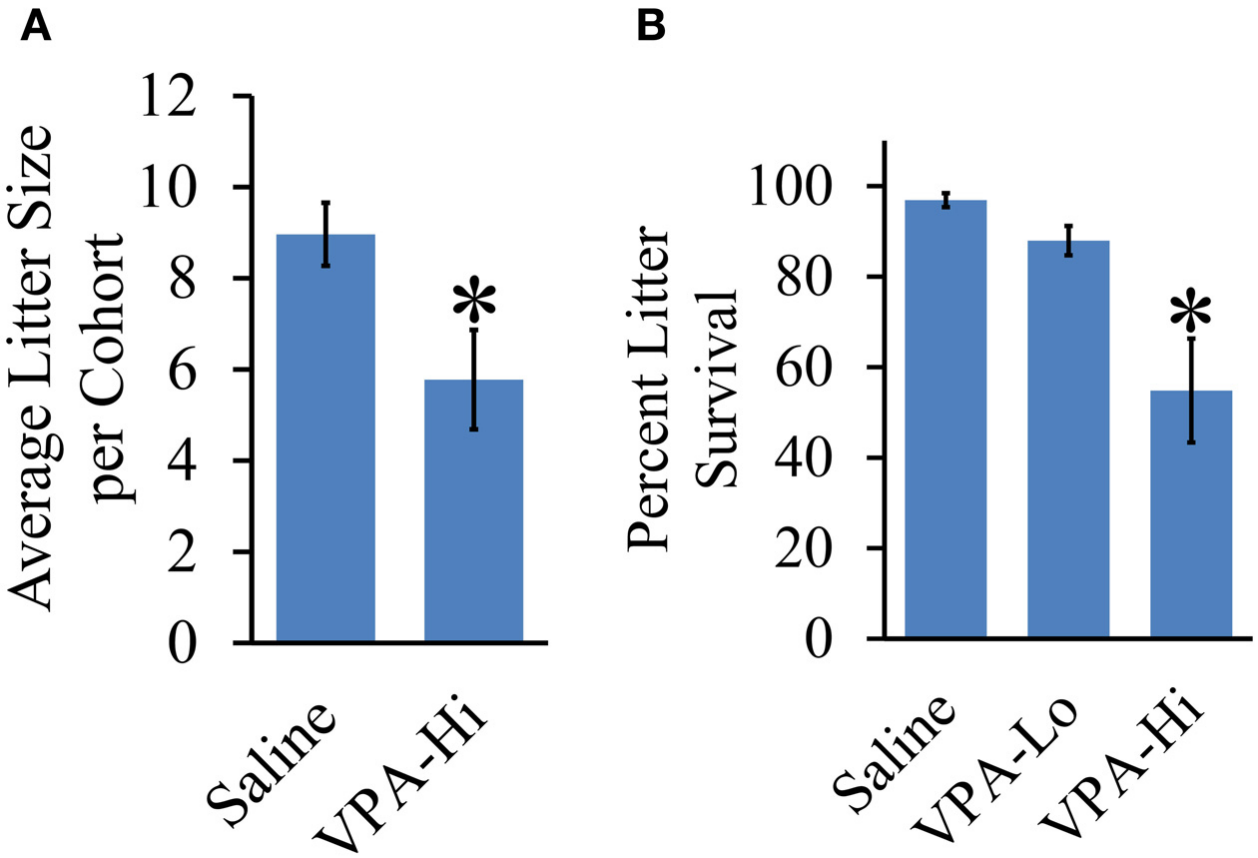
**Effect of VPA exposure *in utero* on average litter size and percent litter survival. (A)** VPA-Hi animals exhibited a significant reduction in average litter size per cohort in comparison to saline animals. **(B)** The percent litter survival was reduced significantly in VPA-Hi animals compared to saline and VPA-Lo animals. No significant difference in percent litter survival was observed in VPA-Lo animals compared to saline animals. Data presented as mean ± s.e.m. (^*^p < 0.05).

We measured the effect of *in utero* VPA exposure on the overall litter survival rate and litter size. Across cohorts of animals, the average litter size was consistently lower for VPA-Hi treated animals compared to saline animals [*t*_(10)_ = 2.52, *p* = 0.03] (Figure 7A). Prenatal exposure to VPA also induces a significant reduction in litter survival rate. A One-Way ANOVA revealed an overall group effect of VPA on litter survival rate [*F*_(2, 22)_ = 9.72, *p* = 0.001]. Fisher's *post-hoc* test revealed significant reduced litter survival rate in VPA-Hi animals compared to saline animals (*p* = 0.0006) and VPA Lo animals (*p* = 0.01) (Figure 7B). These findings are consistent with observations previously reported (Favre et al., 2013) and underscore that there is a bona fide biological difference between the 500 mg/kg (VPA-Lo) and 600 mg/kg (VPA-Hi) doses of VPA.

## Discussion

Our findings that progeny from VPA exposed dams exhibit increased anxiety and altered emotional learning complement and expand upon previous studies from other laboratories. In particular, we provide novel data that indicate that progeny from VPA-Hi exposed dams exhibit significantly impaired acquisition of auditory fear conditioning. These findings are interesting, in part because the VPA-Lo exposed animals do not exhibit this reduction in fear learning. Given that these two rather similar doses of VPA result in different behavioral phenomena and differences in litter sizes and pup survivability, it seems likely the VPA-Hi dose is at or near an important biological tipping point. Notably, a previous study that examined different behavioral phenomena then we have, reported a differential influence on learning using slightly lower (470 mg/kg VPA) and higher (720 mg/kg VPA) doses of VPA, where the lower dose enhanced learning and the higher dose inhibited learning (Frisch et al., 2009). A human population study revealed a significant relationship between the dosage of VPA and human teratogenicity, with higher doses associated with greater risk for birth defects (Vajda et al., 2004). Anatomical defects were also observed in progeny of VPA exposed dams to be dose dependent (Binkerd et al., 1988). Collectively, these results are consistent with our findings.

Most of our findings are largely consistent with the prior published literature; however there are few important differences that are worth highlighting. In our study, we detected a trend, (*p* = 0.13) for enhanced learning during the training phase of fear conditioning, but did not detect differences in memory strength during the testing phases of fear conditioning for animals exposed to VPA-Lo. However, a few prior studies reported enhanced fear learning in VPA-Lo exposed animals. Intriguingly though, these studies report slightly different fear learning phenomena due to VPA-Lo exposure. For example one study found no difference in fear learning for VPA-Lo animals during the training phase of fear conditioning, but found enhanced freezing to conditioned tones during the LTM test, which the authors interpreted as stronger fear memory (Markram et al., 2008). However, it remains possible that the enhanced freezing could be due to either stronger consolidated fear memory or it could be due to reduced intra-trial fear memory extinction. Another laboratory detected enhanced fear learning for VPA-Lo animals during the training phase of fear conditioning, but their LTM data do not unambiguously indicate if the fear memory is stronger or if the animals are simply extinguishing the fear memory faster (Lin et al., 2013; Wang et al., 2013). Considering that the fear learning data from VPA-Lo exposed animals is not consistent across labs, the VPA-Lo induced enhancement of fear learning may be a less robust phenomenon as compared to the VPA-Lo induced fear extinction deficit. For example, the finding that progeny from VPA-Lo exposed dams exhibit reduced fear memory extinction is supported by all prior studies and this study. A different study reported that VPA-Hi exposed animals exhibit enhanced learning for trace and delay fear conditioning (Sui and Chen, 2012). While it is evident that this prior study did not detect an impairment in learning, similar to what we have, it is less clear if the data reported in this prior study data truly reflect an enhancement in delay fear conditioning, since they do not find differences in freezing levels during conditioned tone presentation during STM and LTM testing between VPA and saline exposed animals. This prior study also reported that there was no difference in litter sizes and pup survivability between saline and VPA-Hi treated animals which is incongruent with our study and a prior study (Favre et al., 2013). Considering our study and the above mentioned studies utilized different fear conditioning parameters, fear memory testing parameters and in some cases different strains of rats and age of rats, it is certainly possible that we simply observed slightly different phenomena. Notably differences in VPA induced effects have been found to be dependent on genetic background (Downing et al., 2010), the age at which the VPA exposed animals were examined (Martin and Manzoni, 2014) and maternal stress (Ogawa et al., 2007).

Because auditory fear conditioning could be influenced by impairments in the perception of the pain normally induced by the foot shock, and impairments in hearing of the tone used to auditory fear condition the animals, we examined foot shock/pain sensitivity and hearing to the tone used to auditory fear condition the animals. Our data indicate that progeny from VPA-Hi and saline exposed dams did not exhibit significant differences in these sensory modalities, indicating that differences in fear conditioning between these groups are unlikely due to abnormalities in these sensory modalities. Others have previously found that VPA exposed animals exhibit subtle differences in pain sensitivity (Schneider and Przewlocki, 2005). However, one of the studies that reported these observations, examined thermal pain sensitivity and importantly these animals were not impaired enough in pain sensitivity for it to significantly alter foot shock induced fear conditioning (Markram et al., 2008).

VPA is one of the most widely prescribed drugs for the treatment of epilepsy. The mechanism by which VPA induces teratogenicity is currently not fully understood. Various biochemical studies indicate that VPA can suppress neuronal activity by blocking sodium and calcium channels and enhance the functioning of the inhibitory neurotransmitter, GABA, in the brain (Kwan et al., 2001; Gould et al., 2004). Further, studies have demonstrated the ability of VPA to alter gene expression *in vitro* and *in vivo* (Jergil et al., 2009; Kultima et al., 2010; Cohen et al., 2013; Oguchi-Katayama et al., 2013) and these affects have been attributed to VPA's ability to inhibit histone deacetylase (HDAC) (Phiel et al., 2001; Eyal et al., 2004; Haberland et al., 2009). HDAC plays an important role in regulating transcription during fetal development (Shaked et al., 2008; Montgomery et al., 2009). Consequently, HDAC inhibition may induce abnormal gene expression during embryogenesis, causing behavioral impairments at later time points (Phiel et al., 2001). Notably, a recent rodent study demonstrated that inhibition of HDAC *in utero* is sufficient to cause autism-like phenotypes including sociability deficits in exposed offspring (Moldrich et al., 2013).

Only a few studies have examined the process of fear conditioning among human ASD patients. Gaigg and Bowler (2007) observed that patients diagnosed with Asperger's syndrome displayed attenuated fear conditioning. Specifically, these individuals exhibited reduced skin conductance to conditioned aversive stimuli but normal responses to unconditioned stimuli compared to healthy controls. Another study found that the degree of social impairment observed in ASD individuals directly correlated to their ability to be fear conditioned, i.e., an individual with reduced ability to be fear conditioned had a higher level of social impairment and vice versa (South et al., 2011). It was also found that ASD individuals exhibited delayed reversal learning when a previously conditioned safety cue was changed to be a predictor of aversive stimuli (South et al., 2012). However, all ASD patients may not exhibit deficits in fear conditioning since one study observed that ASD individuals exhibited normal fear learning when examined using a fear potentiated startle response paradigm (Bernier et al., 2005). Nevertheless, the fact that similar fear conditioning deficits were observed in progeny from VPA-Hi exposed dams and a subpopulation of human ASD strongly advocates that further research is needed. Considering that ASD is a very heterogeneous disorder that likely has multiple causes, it could be useful to categorize these patients into distinct entities to facilitate the identification of the neurobiological mechanisms that underlie ASD. For example, it may be possible to screen ASD individuals for their ability to be fear conditioned and use this metric as a biomarker or endophenotype that could help categorize ASD individuals and thus serve as a way to disambiguate the heterogeneous nature of this disorder.

## Author contributions

Anwesha Banerjee, Bethany L. Sauls and Anna A. Morales have bred and maintained the animal colonies and litter details including litter size and litter survival within this study. Anwesha Banerjee performed the fear conditioning, open field, sensory modalities and social interaction experiments. Anwesha Banerjee, Bethany L. Sauls, Anna A. Morales performed the object recognition tests. Bethany L. Sauls, Anna A. Morales performed blind data scoring for foot shock sensitivity and paw lick test. Crystal T. Engineer and Michael P. Kilgard have conceived and carried out the tone threshold experiment. Anwesha Banerjee and Jonathan E. Ploski conceived the study and participated in its design and coordination and drafted the manuscript. All authors read, edited and approved the final manuscript.

### Conflict of interest statement

The authors declare that the research was conducted in the absence of any commercial or financial relationships that could be construed as a potential conflict of interest.

## Supplementary material

Table S1 **The table depicts the number of litters per cohort of animals per treatment, with each experiment and order of experiments performed on each cohort of animals listed**.

Image 1 **Progeny from VPA-Hi exposed dams exhibit deficits in auditory fearing conditioning**. Progeny from VPA-Hi and saline exposed dams were auditory fear conditioned (Cohort 1: Saline = 8, VPA-Hi = 7, Cohort 2: Saline = 8, VPA-Hi = 6. **(A)** Pre and post-shock freezing was assessed immediately before and after exposure to tone-shock pairing in VPA-Hi cohort 1 animals. **(B)** Auditory fear memory assessed 24 h post fear conditioning (i.e., LTM) in VPA-Hi cohort 1 animals. **(C)** Pre and post-shock freezing was assessed immediately before and after exposure to tone-shock pairing in VPA-Hi cohort 2 animals. **(D)** Auditory fear memory assessed 24 h post fear conditioning in VPA-Hi cohort 2 animals (i.e., LTM). Bars represent the mean ± standard error of the mean (SEM) (^*^*p* < 0.05).
