# Supplementary material for: Abnormal emotional learning in a rat model of autism exposed to valproic acid in utero
Source: Front Behav Neurosci. 2014 Nov 12;8:387. doi: 10.3389/fnbeh.2014.00387 (PMC4228846; doi:10.3389/fnbeh.2014.00387)
Supplement: Table S1 — The table depicts the number of litters per cohort of animals per treatment, with each experiment and order of experiments performed on each cohort of animals listed. [file Table1.PDF]

**Cohort**	**Treatment, # litters**	**Experiment 1**	**Experiment 2**	**Experiment 3**
Cohort 1	Saline, 2 litters	Open Field	Fear Conditioning	
Cohort 1	VPA-Hi, 3 litters	Open Field	Fear Conditioning	
Cohort 2	Saline, 2 litters	Open Field	Fear Conditioning	
Cohort 2	VPA-Hi, 3 litters	Open Field	Fear Conditioning	
Cohort 3	Saline, 4 litters	shock threshold		
Cohort 3	VPA-Hi, 6 litters	shock threshold		
Cohort 4	Saline, 4 litters	Open Field	Object Recognition	Paw lick
Cohort 4	VPA-Hi, 5 litters	Open Field	Object Recognition	Paw lick
Cohort 5	Saline, 5 litters	Social Interaction		
Cohort 5	VPA-Hi, 7 litters	Social Interaction		
Cohort 6	Saline, 4 litters	A1 spiking firing measurement		
Cohort 6	VPA-Hi, 7 litters	A1 spiking firing measurement		
Cohort 7	Saline, 5 litters	Open Field	Fear Conditioning	shock threshold
Cohort 7	VPA-Lo, 7 litters	Open Field	Fear Conditioning	shock threshold
Cohort 8	Saline, 4 litters	Open Field	Object Recognition	Paw lick
Cohort 8	VPA-Lo, 5 litters	Open Field	Object Recognition	Paw lick
