# Supplementary figures and images for: Abnormal emotional learning in a rat model of autism exposed to valproic acid in utero
Source: Front Behav Neurosci. 2014 Nov 12;8:387. doi: 10.3389/fnbeh.2014.00387 (PMC4228846; doi:10.3389/fnbeh.2014.00387)

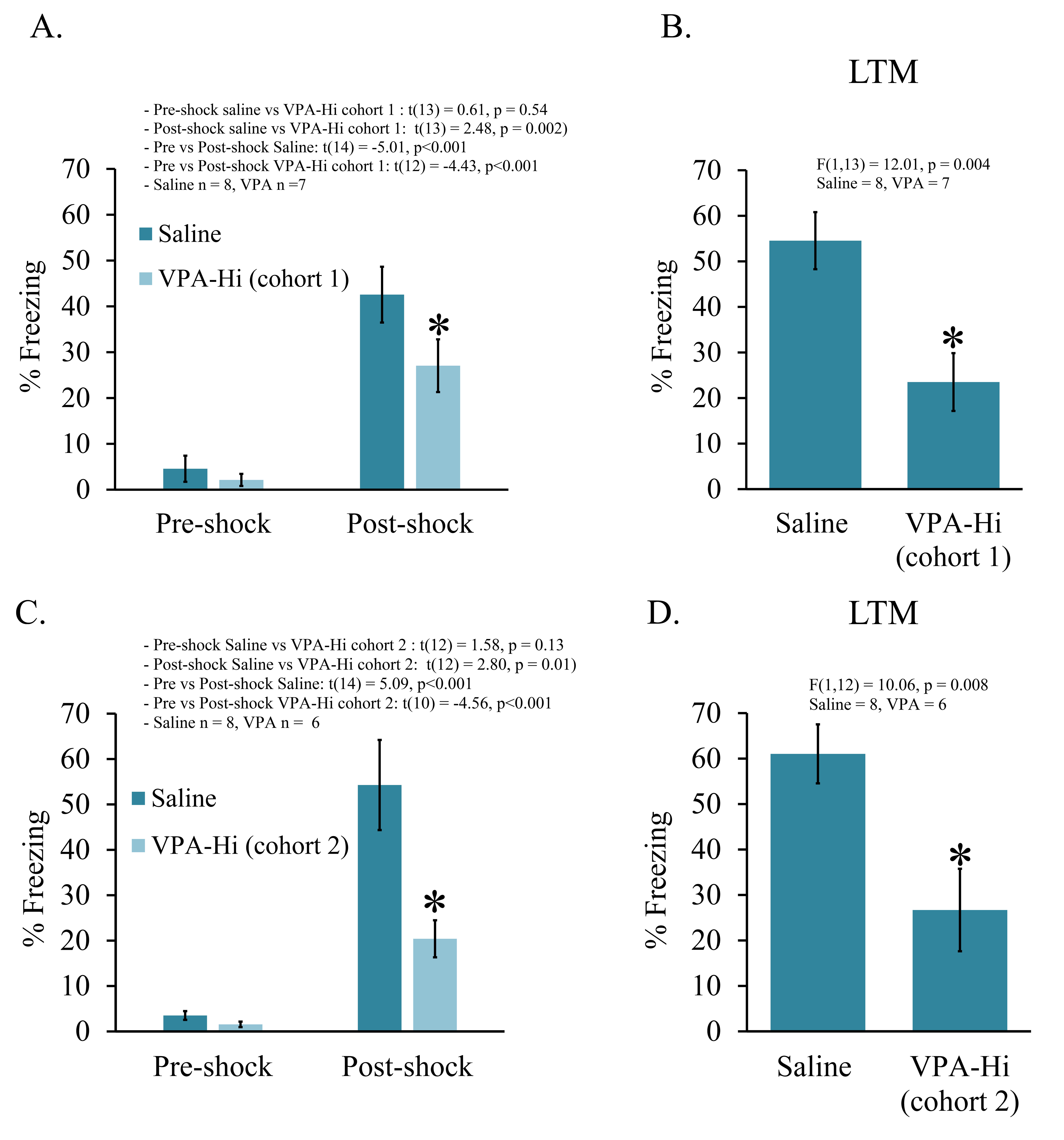

Supplement: Image 1 — Progeny from VPA-Hi exposed dams exhibit deficits in auditory fearing conditioning. Progeny from VPA-Hi and saline exposed dams were auditory fear conditioned (Cohort 1: Saline = 8, VPA-Hi = 7, Cohort 2: Saline = 8, VPA-Hi = 6. (A) Pre and post-shock freezing was assessed immediately before and after exposure to tone-shock pairing in VPA-Hi cohort 1 animals. (B) Auditory fear memory assessed 24 h post fear conditioning (i.e., LTM) in VPA-Hi cohort 1 animals. (C) Pre and post-shock freezing was assessed immediately before and after exposure to tone-shock pairing in VPA-Hi cohort 2 animals. (D) Auditory fear memory assessed 24 h post fear conditioning in VPA-Hi cohort 2 animals (i.e., LTM). Bars represent the mean ± standard error of the mean (SEM) (*p < 0.05). [file Image1.JPEG]
